# The Impact of Different Types of Auditory Warnings on Working Memory

**DOI:** 10.3389/fpsyg.2022.780657

**Published:** 2022-02-25

**Authors:** Zhaoli Lei, Shu Ma, Hongting Li, Zhen Yang

**Affiliations:** Department of Psychology, Zhejiang Sci-Tech University, Hangzhou, China

**Keywords:** auditory warnings, auditory icons, earcons, spearcons, working memory, interference

## Abstract

Auditory warnings have been shown to interfere with verbal working memory. However, the impact of different types of auditory warnings on working memory tasks must be further researched. This study investigated how different kinds of auditory warnings interfered with verbal and spatial working memory. Experiment 1 tested the potential interference of auditory warnings with verbal working memory. Experiment 2 tested the potential interference of auditory warnings with spatial working memory. Both experiments used a 3 × 3 mixed design: auditory warning type (auditory icons, earcons, or spearcons) was between groups, and task condition (no-warning, identify-warning, or ignore-warning) was within groups. In Experiment 1, earcons and spearcons but not auditory icons worsened the performance on the verbal serial recall task in the identify-warning condition, compared with that in the no-warning or ignore-warning conditions. In Experiment 2, only identifying earcons worsened the performance on the location recall task compared with performance without auditory warnings or when auditory warnings were ignored. Results are discussed from the perspective of working memory resource interference, and their practical application in the selection and design of auditory warning signals is involved.

## Introduction

Auditory warnings include speech and non-speech sounds. Speech auditory warnings are mainly used to display content information and are widely applied in multimedia interfaces, telephone communication systems, vehicle systems, medical treatment, and special populations with blind or low eyesight. However, their usage is limited by poor confidentiality and slow processing speed due to the need to listen to the full sentence to understand the meaning. By contrast, non-speech sounds are preferred due to privacy concerns or in situations where specific speech prompts are not required (Isherwood and McKeown, 2017). Compared with speech auditory warnings, non-speech auditory warnings have better confidentiality, speech independence, and wide applicability in different countries and dialects.

### Auditory Icons, Earcons, and Spearcons (Speech-Based Earcons)

Common representations of non-speech interfaces mainly include auditory icons and earcons. Auditory icons are sounds used to represent their associated events or attributes in daily life (Gaver, 1989), which refer to conveying computer operations or events by imitating familiar sounds of real-world events. They are usually relatively brief and icon-like (Larsson and Niemand, 2015; Amer and Johnson, 2018). For example, the sound of a broken plate is used to represent the operation of deleting a file, and the sound of a dot-matrix printer or typewriter signifies a printing operation.

Earcons are abstract short non-verbal auditory information with musical nature used to provide information and feedback on computer operations or interactions (Blattner et al., 1989; Brewster et al., 1993; Amer et al., 2013; Larsson and Niemand, 2015). For example, the rising “login” melody and the descending “logout” melody in the Windows operating system are formed by different combinations of high and low tones. Earcons can be mapped to any object, operation, or interaction event, and are designed as a series of mappings to represent hierarchical structure by manipulating their parameters, such as timbre and pitch (Garzonis et al., 2009).

To compensate for the weaknesses of traditional non-speech auditory cues, researchers developed spearcons, a compromise between short non-speech stimuli and full speech stimuli. These signals are short, time-compressed spoken words or speech phrases that are sped up even to the point where they are no longer considered speech. Spearcons can directly and quickly convey their meaning and relevant information to the listener (Petocz et al., 2008; Walker et al., 2013; Jeon, 2015), have good learnability, and can remarkably improve the efficiency and accuracy of menu navigation search (Palladino and Walker, 2007; Dingler et al., 2008; Walker et al., 2013). Compared with earcons, spearcons can provide a direct mapping between sounds and menu items, flexibly covering more content domains, and thus having better flexibility and generation. Therefore, spearcons have been studied and applied in some fields, such as patient monitoring alarms and menu navigation (Walker et al., 2013; Li et al., 2017; Sanderson et al., 2019).

### Potential Hazards of Auditory Warnings and Their Impact on Working Memory

Auditory warnings have become ubiquitous in daily work environments. Although they improve the efficiency of human-computer interaction, their potential hazards must be considered. First, the environment in which auditory warnings are used may require a high degree of concentration of the operator. Despite conveying important information, these signals might be not necessarily urgent. In addition, not every warning sound is important or urgent for every operator in the same environment. Given that sound signals are omnidirectional and forced hearing, people always easily get distracted and drawn to sounds that are not relevant or meaningful at the moment, even when they try to focus on something important (Banbury et al., 2001; Watson et al., 2004). When the alarm sounds, the irrelevant operator may be engaged in a cognitively demanding task, such as driving, intensive care, or surgery. Once attracted to the sound information, the operator may not focus on the important tasks. This situation may create some potential hazards. It has been found that an auditory warning of a relatively small event may lead to errors in the input of coordinates in navigation or weapon delivery systems, resulting in potentially serious consequences (Banbury et al., 2001). Furthermore, Lacherez et al. (2016) mentioned that auditory warning identification may compete with other cognitive processes for working memory resources and result in poor performance on other tasks. Many situations relying on auditory display assistance are related to user’s defects in performing dual tasks, such as in patients with Parkinson’s disease (Ashburn et al., 2001) or head injuries (Hart et al., 2002; Hein et al., 2005). Ashburn et al. (2001) found that patients with Parkinson’s disease who are prone to fall also perform poorly in dual tasks. Hence, auditory warnings and auxiliary systems must aim to control the specific cognitive demands (Ashburn et al., 2001), to avoid additional negative impacts on users or reduce the availability of systems.

Cognitive tasks, which usually rely on a person’s working memory, have been increasingly involved in many human activities. The impact of auditory warnings on the operator’s task performance is mainly concentrated on the working memory. Working memory refers to a memory system with limited capacity for temporary processing and storage of information (Baddeley, 2003). It plays an important role in many complex cognitive activities. Many theoretical models have attempted to explain this memory system. One that is widely held is the Baddeley’s Working Memory Model (Baddeley and Hitch, 1974), which suggests that working memory consists of visuo-spatial sketchpad, phonological loop, and central executive. Later research came up with the episodic buffer, forming the four-component model of the working memory system (Baddeley, 2000b).

Auditory information may interfere with working memory in a complex task environment. For example, those with changing patterns interfere with serial recall task performance. Irrelevant sounds (e.g., the sounds do not need to be noticed) can also interfere with the current task (Banbury et al., 2001; Macken and Jones, 2003; Hughes et al., 2007; Macken et al., 2009), and this phenomenon is called “Irrelevant Sound Effect (ISE).” Using the ISE paradigm, researchers found that the accuracy of reports decreased by 30–50% when unrelated narrative statements were played during a serial recall task (Ellermeier and Zimmer, 1997). Experimental analysis on the effect of external cockpit sounds on crew performance showed that compared with quiet or ambient aircraft noise, the presence of external background sounds substantially disrupted the memory of longitude and latitude information by up to 60% (Banbury and Jones, 1999). Serial recall was also hampered by various non-speech sounds, including pure tones (e.g., Klatte et al., 1995; Neath et al., 1998) and music streams (e.g., Nittono, 1997). Moreover, the interference of sound may be stable and difficult to be habituated (Jones et al., 1997; Tremblay and Jones, 1998), even if prolonged exposure did lead to some degree of habituation, and relatively short quiet periods could drive rapid dishabituation (Banbury and Berry, 1998).

Further research revealed that the perception and identification of learned auditory warnings can also interfere with working memory. However, learned melody and rhythm auditory warnings would interfere only when the participants attempt to identify them. By contrast, learned non-word phrases would interfere even when ignored (Lacherez et al., 2016). Given their different characteristics, we speculated that various kinds of auditory warnings may interfere with working memory differently. Alarm sounds used in previous studies were either earcons (e.g., rhythm and melody) or spoken non-word phrases. The impact of auditory icons and spearcons on working memory has not been determined. Spearcon is a hybrid auditory display between speech and non-speech (Jeon, 2015), and it appears to have both verbal and non-verbal attributes. Researchers have found that concurrent verbal tasks had a negative impact on the identification of spearcons (Davidson et al., 2019), and identifying learned spearcons may interfere with speech-based working memory tasks (Wolters et al., 2012). However, the impact of ignoring spearcons and auditory icons on working memory has not been explored, and no research has compared the interference of different kinds of auditory warnings on working memory.

### Relevant Theoretical Models: Impact of Auditory Warnings on the Different Domains of Working Memory and the Mechanism

It is widely accepted that working memory system is divided into verbal and spatial working memory. Most previous studies have focused on the impact of auditory warnings on verbal working memory. However, the influence of auditory warnings on spatial working memory and whether they interfere differently with the two domains deserve further exploration.

Due to the forced hearing nature of the sound signal, the warning sound tends to attract people’s attention. When the alarm sounds, some operators in the workplace may need to ignore it, but it may still be distracting or interfere with working memory. In the cognitive behavioral tradition, studies on the mechanism of sound interference with working memory performance have been mainly focused on how working memory task is interfered with by unrelated sounds that change acoustically (i.e., the changing-state effect) (Jones et al., 1992; Lecompte, 1995), and the physiological and behavioral distraction effect of an auditory event that deviates in some way from the recent hearing (i.e., the deviation effect) (Cowan, 1995; Titova and Näätänen, 2001). The duplex-mechanism account holds that sound can cause unnecessary auditory distraction either by interfering specifically with the processes involved in the focal task (interference-by-process) or by diverting attention away from a focal task regardless of the type of processing involved in the task (attentional capture) (Hughes et al., 2007; Hughes, 2014). In this view, the changing-state effect can be better explained by recourse to interference-by-process, and the deviation effect may be attributed to attentional capture. In another case, and in most cases, operators may need to identify warnings and determine mentally whether they need to take corresponding actions. There may be a distraction problem both when ignoring and when identifying the warnings. Whether distraction (or switching attention) and the process of identifying warnings would affect ongoing tasks involving verbal and spatial working memory may be related to resource limitation and interference.

Multiple Resource Theory (MRT) proposes four important categorical and dichotomous dimensions that account for variance in time-sharing performance. Each dimension has two discrete “levels,” each defining a separate but limited resource. The four dimensions are processing stages (perception and cognition vs. selection and response), perceptual modalities (auditory vs. visual), visual channels (focal vs. ambient), and processing codes (spatial vs. verbal) (Wickens, 2002). MRT predicts that resource interference occurs when two tasks are performed using the same domain resources, and worsens the performance compared with that when using different domain resources. For example, the interference between two tasks both requiring verbal perception is greater than that between one task requiring spatial perception and the other requiring verbal perception. What is noteworthy is that regardless of doing one or two tasks, MRT is relevant only in the region where overload is imposed by multiple tasks but not in the residual capacity region. For example, it can predict the size of dual-task decrements once overload has been reached (Wickens, 2008).

Similarly, the multi-resource model of working memory also involves the domain-specific assumptions about limited resources: working memory consists of multiple domain-specific subsystems, and each subsystem has its own resource pool (e.g., Baddeley and Logie, 1999). The nature of resources is domain-specific, that is, specific resources support verbal or visuospatial activities. Therefore, interference occurs when the two tasks involve information belonging to the same domain, and no (or minimal) interference occurs when the tasks involve information belonging to different domains. Verbal working memory is more likely to be interfered with by verbal tasks than by spatial tasks, and spatial working memory is more susceptible to interference from spatial tasks than from verbal tasks (Vergauwe et al., 2010).

In addition, another assumption about limited resources is that a general limited resource pool supports various cognitive activities (e.g., Egeth and Kahneman, 1975; Barrouillet et al., 2004). This pool of resources is often called attention. Verbal and spatial activities are assumed to compete for a common pool of domain-general limited resources, resulting in interference between the two activities (Vergauwe et al., 2010). Banbury and Jones (1999) found that speech interfered with visuospatial task performance despite being ignored. Studies have further confirmed that verbal and spatial activities interfered with each other under dual-task conditions, indicating the existence of a domain-general resource in the mental process of verbal and spatial (Vergauwe et al., 2010; Morey et al., 2018). Mobile phone use impaired driving safety, regardless of whether the phone was hand-held or hands-free (Strayer and Johnston, 2001). This finding suggests that processing sound information interferes with spatial tasks at least to a certain extent. However, most of the concurrent tasks in previous studies were verbal tasks (e.g., speech or text). The impact of non-speech auditory displays on spatial working memory remains to be further clarified.

This study attempted to explore and explain the impact of auditory warnings on working memory and its mechanism based on the duplex-mechanism account and the related resource theories. Based on the review of relevant literature, how identifying and ignoring three types of auditory warnings (auditory icons, earcons, and spearcons) affects performance on a verbal serial recall task (i.e., spatial working memory), and whether there are differences among them have not been determined. We investigated these questions in Experiment 1. We hypothesized that warning identification would have more influence on recall task performance compared with warning ignoring, and different types of auditory warnings would worsen recall task performance differently. Furthermore, we further explored whether the performance of location recall task (i.e., spatial working memory) was similarly affected by the three types of auditory warnings in Experiment 2. According to related theories, we hypothesized that auditory warnings would worsen the performance of location recall task, and the three types of auditory warnings impact location recall differently. Overall, this study evaluated the impact of different types of auditory warnings on the performance of verbal and spatial working memory tasks. The findings may help to draw people’s attention to the potential problems of using auditory warnings in related environments, especially those that require high working memory load, and may serve as a caution against the possible existence of overuse of auditory warnings in such environments. In addition, the impact of three types of auditory warnings (auditory icons, earcons, and spearcons) on working memory was investigated to provide useful guidelines for the selection and design of auditory warning signals. Finally, the differences in the interference degree of auditory warnings for verbal and spatial working memory were also analyzed.

## Experiment 1

Experiment 1 aimed to test the impact of different types of auditory warnings on verbal working memory.

### Materials and Methods

#### Participants

Seventy-two participants aged 17–25 years (*M* = 19.33, *SD* = 2.02), including 37 females and 35 males completed the study. The number of participants was determined by using G*Power. The statistical power (1−β) is function of the type I error (α = 0.05), power was set to 0.80, power analysis was conducted for a medium effect size (*f* = 0.25). Analysis indicated that to detect a medium effect size would have required 69 participants. Considering that the number of participants required for the Latin square design of task conditions was a multiple of 6, a total of 72 participants were recruited. All participants were recruited from Zhejiang Sci-Tech University and were paid CNY20 (US$3) as compensation for their time. The participants were randomly divided into three groups of 24 members, and all individuals reported normal visual (or corrected vision) and normal hearing.

#### Apparatus and Materials

The experimental program was written and conducted by E-Prime version 3.0 and presented on the 13.3-inch laptop monitor. All sounds were presented through the Sennheiser HD206 stereo headset, and the volume was set at a comfortable level (approximately between 30 and 36%) for the participants. Serial recall tasks were used for the testing period, and the participants were instructed to simulate the monitoring of chemical reactions (see Lacherez et al., 2016, for a similar recall task).

Auditory warnings were grouped into auditory icons, earcons, and spearcons with four warnings each. The length of warnings was between 903 and 1,078 milliseconds. The material of the auditory icons was taken from the ear0.com website and used after cropping, noise reduction, and fade-in and fade-out settings. The four auditory icons were as follows: the sound of pouring and gradually filling water in a cup represented the concentration imbalance warning; the sound of a ship horn represented the warning of volume imbalance; the sound of hot water boiling represented the warning of temperature imbalance; and the sound of glass bursting represented the pressure unbalance warning.

Rhythm alarms used in previous studies (Lacherez et al., 2016) were used for the earcons. The rhythm alarms were composed of four tones, each of the same note value, varying in length and in four different arrangements. These rhythm alarms were properly cropped and compressed without changing the pitch to keep the length within the range of 903–1,078 milliseconds by using GoldWave 6.41.

Spearcons in this study were generated by compressing the TTS phrases. TTS items were linearly time-compressed to between 30 and 40% of their original length while maintaining original pitch. Eighteen volunteers were recruited to complete a questionnaire survey on the semantic recognition of spearcons, and 88.89% of the volunteers thought that the final spearcons could not be recognized as a specific speech. Therefore, we regarded that these spearcons satisfied the definition of “the spoken phrases are sped up even to the point where they are no longer considered speech.”

#### Design

In Experiment 1, a 3 (auditory warning type) × 3 (task condition) mixed design was used. Participants were randomly assigned to one of three experimental groups: auditory icon, earcon, or spearcon groups. The participants in each group only heard the named auditory warning type in identify-warning and ignore-warning conditions. They completed the serial recall task once in the no-warning condition, once in the identify-warning condition, and once in the ignore-warning condition. The dependent variables were serial recall accuracy (the answer was recorded as correct when all eight digits were correctly recalled in the order presented) and warning identification accuracy (available only in the identify-warning condition).

##### No-Warning Condition

In this condition, the participants performed the serial recall task without any auditory warnings and were shown standard instructions on the screen prior to the test. After two practice trials, the individuals started the formal recall task and completed 24 serial recall trials. Each trial consisted of eight digits presented in a random order without repetition, and each digit appeared on screen for 800 milliseconds. The participants were required to remember all eight digits in the order of appearance. At the conclusion of the eight-digit presentation, a blank screen was shown for 2 s before the response box appeared, and the participants then recalled and entered their response in the box by tapping the keyboard. A response was scored as correct only when the eight digits were repeated correctly in the order presented. After the digits were completely inputted, the participants were cued for the beginning of the next trial.

##### Identify-Warning Condition

In this condition, the participants conducted two practice trials and then completed 24 serial recall trials after being presented with standard instructions. The eight digits were presented in the same way as the no-warning condition; however, during such time the participants were interspersed once with one of the auditory warnings that they had learned in the learning period. The warning appeared randomly between the first and second digits, the third and fourth digits, the fifth and sixth digits, or the seventh and eighth digits. An identical blank screen of 2 s occurred at the end of the eight-digit presentation. After the participants entered their serial recall response, an identification screen appeared in which they were instructed to identify the auditory warning by pressing a specific key on the keyboard, as they had done in the learning period, and then proceed to the next trial.

##### Ignore-Warning Condition

The ignore-warning condition was identical to the identify-warning condition except those participants were told to ignore the presented auditory warning and were not required to identify and respond to it at the end.

#### Procedure

Prior to the experiment, the relevant demographic information of the participants was collected, and the structural process of the experiment was briefly described. Participants read the necessary instructions presented on the screen and individually completed the experiment in a quiet laboratory. They underwent a learning session to master a set of four warnings (either auditory icons, earcons, or spearcons), which were then presented while the participants were engaged in a serial recall task in the testing period. Participants completed the auditory warnings learning period before starting the formal testing period.

##### Learning Period

Participants in each group underwent a learning period to learn an association between an auditory warning and a response. Four distinct warnings were used for each group. Participants were instructed to monitor a chemical reaction, and each individual auditory warning represented an imbalance in either the concentration, volume, temperature, or pressure of the reaction. This process aimed to avoid any association with any existing warnings that are familiar to the participants and create a generic semantic association with an arbitrary quantity (Lacherez et al., 2016). In the initial phase of the learning period, each auditory warning and its related parameter were presented together three times for 1,200 ms each. Participants then underwent a testing phase in which the warnings were presented individually in random order. They were asked to identify the warning by entering a specified key of the parameter it represented (four stickers on the keys indicated the parameters of the warnings: F for concentration, J for temperature, V for volume, and N for pressure). Participants were given feedback on the accuracy of their responses. Each auditory warning was presented three times, and the individuals were considered to have learned and ended the testing when they got all 12 correct answers; otherwise, they had to repeat the testing until they reached 100% accuracy.

##### Testing Period

Each participant completed the digital serial recall tasks once in every condition. The three task conditions were counterbalanced following the Latin square design. Participants practiced two trials before each task to ensure that they understood the procedure of the task. At the conclusion of each block of 24 trials, the participants were invited to take a short break (for approximately 2 min) before proceeding to the next phase.

The duration of the entire experiment was approximately 40 min.

### Results

Mauchly’s test of sphericity was examined, and the Greenhouse-Geisser correction was used where necessary. Descriptive statistics of the mean serial recall accuracy of auditory icon, earcon, and spearcon groups in different task conditions are shown in Table 1.

**TABLE 1:** *Experiment 1:* Mean serial recall accuracy (%) in the no-warning, identify-warning, and ignore-warning conditions for the auditory icon, earcon and spearcon groups.

	No-warning *M* (*SD*)	Identify-warning *M* (*SD*)	Ignore-warning *M* (*SD*)
Auditory icon	86.46 (12.96)	87.15 (14.84)	90.80 (7.37)
Earcon	88.20 (8.66)	69.97 (22.66)	88.19 (10.55)
Spearcon	85.42 (12.59)	66.84 (21.01)	84.20 (12.41)
Total	86.69 (11.46)	74.65 (21.48)	87.73 (10.53)

One-way ANOVA was performed on the no-warning baseline scores to ensure comparability among the three groups. The result confirmed that there was no significant difference among the three warning-type groups, *F* (2, 69) = 0.354, *p* = 0.703, partial η^2^ = 0.01.

**FIGURE 1:**
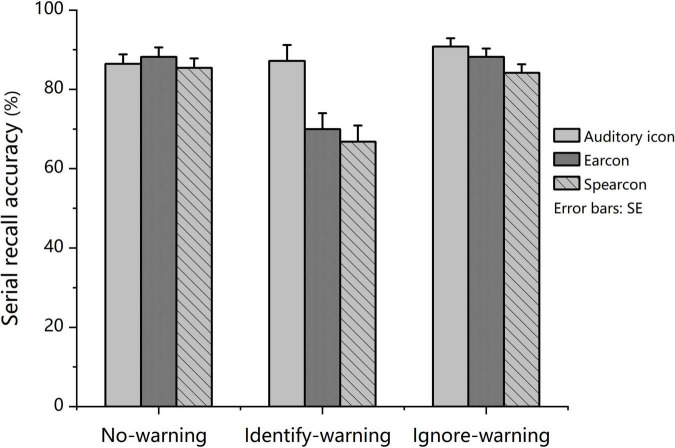
*Experiment 1:* Mean serial recall accuracy in the no-warning, identify-warning, and ignore-warning conditions for the auditory icon, earcon and spearcon groups.

The results from mixed-design 3 × 3 factorial ANOVA showed that both task condition and warning type significantly affected the serial recall accuracy. The task condition revealed a main effect for task performance, *F*(2, 138) = 34.23, *p* < 0.001, partial η^2^ = 0.332, and the main effect of warning type was significant, *F* (2, 69) = 3.922, *p* = 0.024, partial η^2^ = 0.102. In addition to the main effect, a significant two-way interaction was found between task condition and warning type (see Figure 1), *F*(4, 138) = 7.092, *p* < 0.001, partial η^2^ = 0.171.

Further simple effect analysis was conducted by using a *post-hoc* test with Bonferroni correction. The results revealed that for the auditory icon group, no significant difference in mean serial recall accuracy was found among the three conditions. For the earcon and spearcon groups, the mean serial recall accuracy for the identify-warning condition was significantly lower than that for the other two conditions (*p* < 0.001), but no significant difference was observed between the no-warning and the ignore-warning conditions. For the identify-warning condition, the mean serial recall accuracy of the auditory icon group was significantly higher than that of the earcon and spearcon groups (*p* < 0.05). For the ignore-warning condition, there was no significant difference in serial recall accuracy among the three groups.

**FIGURE 2:**
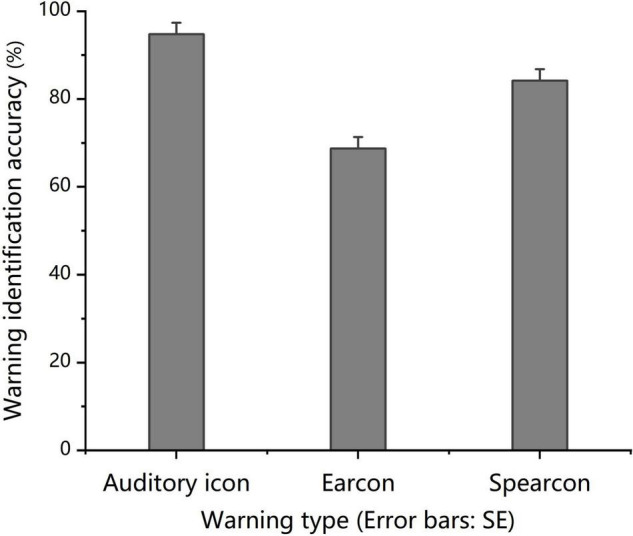
*Experiment 1:* Mean warning identification accuracy in the identify-warning condition for the auditory icon, earcon and spearcon groups.

The one-way ANOVA results revealed significant differences in the mean identification accuracy of the three groups, *F*(2, 69) = 25.311, *p* < 0.001, partial η^2^ = 0.423. The mean identification accuracy for auditory icons was significantly higher than that for earcons and spearcons (*p* < 0.05), and that for spearcons was significantly higher than that for earcons (*p* < 0.001), as shown in Figure 2.

Spearman correlation analysis was conducted on the mean serial recall accuracy and the warning identification accuracy with the learnability (related to the number of practices when reached 100% accuracy) to confirm whether the results of this experiment were related to the learnability. The fewer practices to gain 100% accuracy indicated the better learnability. No significant correlation was found between learnability and serial recall accuracy when the auditory warning required to be identified (*r* = 0.214, *p* > 0.05) or ignored (*r* = −0.015, *p* > 0.05). However, a significant correlation was observed between learnability and mean warning identification accuracy (*r* = 0.451, *p* < 0.001).

### Discussion

The results of Experiment 1 showed that in the identify-warning condition, earcon and spearcon identification worsened the performance on the serial recall task. These results were consistent with previous studies. Identifying spearcons may interfere with verbal working memory tasks (Wolters et al., 2012). The perception and identification of learned earcons (rhythm) interfere with working memory (Lacherez et al., 2016). However, auditory icon identification did not interfere with the performance of the serial recall task. The identification of auditory icons may require less working memory than the identification of earcons and spearcons. Participants may have been performing the identifying auditory icon warnings within their residual capacity of available resources, preserving high accuracy at serial recall tasks (Wickens, 2008).

Auditory icon warnings had the highest warning identification accuracy among the three groups. This finding may be related to the use of sounds from real, daily events in auditory icons, and the fact that these signals are strongly representative and easy to learn. Moreover, it was found in our present study that warning identification accuracy was related to the number of practices (few practices indicated high accuracy). Therefore, we speculated that warning identification accuracy may be related to its learnability. However, the impact on identification performance caused by the resource’s competition of concurrent verbal serial recall tasks could not be ruled out. Furthermore, whether the spatial working memory is similarly affected when the participants identify or ignore the three types of auditory warnings remains unclear. These issues will be addressed in Experiment 2.

## Experiment 2

Experiment 2 aimed to test the impact of different types of auditory warnings on spatial working memory.

### Materials and Methods

#### Participants

Seventy-two participants aged 17–25 years (*M* = 20.01, *SD* = 2.20), including 29 females and 43 males completed this study. The number of participants was determined by using G*Power, and the parameter settings were identical to Experiment 1. Considering that the number of participants required for the Latin square design of task conditions was a multiple of 6, a total of 72 participants were recruited. All participants were recruited from Zhejiang Sci-Tech University and were paid CNY20 (US$3) as compensation for their time. Participants were randomly divided into three groups of 24 members, and all individuals reported normal visual (or corrected vision) and normal hearing.

#### Apparatus and Materials

The apparatus and materials in Experiment 2 were generally similar to those in Experiment 1 except for the verbal tasks being replaced with spatial tasks.

#### Design

A 3 (auditory warning type) × 3 (task condition) mixed design was used in Experiment 2. The participants in each of the auditory icon, earcon, and spearcon groups only heard the named auditory warning type. They completed the red square location recall task (see Vergauwe et al., 2010, for a similar recall task) once in every condition (no-warning, identify-warning, and ignore-warning). The dependent variables were red square location recall accuracy (the answer was recorded as correct when all five red squares were correctly recalled in the order presented) and warning identification accuracy (available only in the identify-warning condition).

##### No-Warning Condition

**FIGURE 3:**
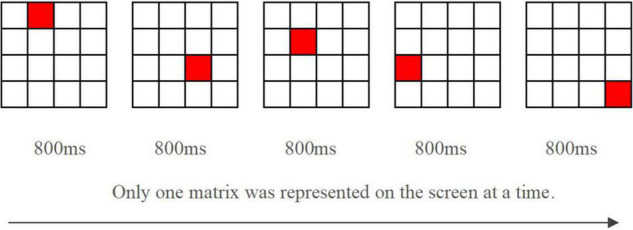
*Experiment 2:* The demonstration of red squares in location recall task (one of the random orders).

In this condition, participants performed the red square location recall task without any auditory warnings. They were shown standard instructions on the screen before beginning the testing. Participants conducted two practice trials and then completed 24 location recall trials. Each trial consisted of a 4 × 4 matrix. Five red squares randomly appeared at different positions in the matrix for 800 milliseconds each (see Figure 3). Participants were required to remember all five positions of the red squares in order. At the conclusion of the five red squares presentation, a blank screen was shown for 2 s before the response box appeared. The participants then recalled and selected correct locations in an empty 4 × 4 matrix by clicking the mouse. After clicking the “submit” button, they were cued for the beginning of the next trial.

##### Identify-Warning Condition

In this condition, participants conducted two practice trials and then completed 24 location recall trials after being presented with standard instructions. The five red squares were presented in a 4 × 4 matrix in an identical way to the no-warning condition. However, during this time participants were interspersed with one of the auditory warnings that they had learned in the learning period, played once. The auditory warning appeared randomly between the first and second matrices, the second and third matrices, the third and fourth matrices, or the fourth and fifth matrices. A blank screen also appeared for 2 s at the end of the five-square presentation. After the location recall task was completed and submitted, an identification screen appeared in which the participants were instructed to identify the auditory warning by pressing a specific key on the keyboard and then proceed to the next trial.

##### Ignore-Warning Condition

The ignore-warning condition was identical to the identify-warning condition except that the participants were told to ignore the presented auditory warning and were not required to identify and respond to it at the end.

#### Procedure

The procedure of Experiment 2 was similar to that of Experiment 1. Each participant completed the learning of their named auditory warning type before starting the formal testing stage. The duration of the entire experiment was approximately 35 min.

### Results

Mauchly’s test of sphericity was examined, and the Greenhouse-Geisser correction was used where necessary. Descriptive statistics of the mean red square location recall accuracy of each group in different task conditions are shown in Table 2.

**TABLE 2:** Mean location recall accuracy (%) in the no-warning, identify-warning, and ignore-warning conditions for the auditory icon, earcon and spearcon groups.

	No-warning *M* (*SD*)	Identify-warning *M* (*SD*)	Ignore-warning *M* (*SD*)
Auditory icon	73.78 (14.56)	72.40 (17.28)	73.96 (19.08)
Earcon	78.30 (15.73)	62.33 (22.20)	76.56 (15.92)
Spearcon	70.31 (17.26)	64.58 (18.06)	71.35 (16.95)
Total	74.13 (16.01)	66.44 (19.52)	73.96 (17.25)

**FIGURE 4:**
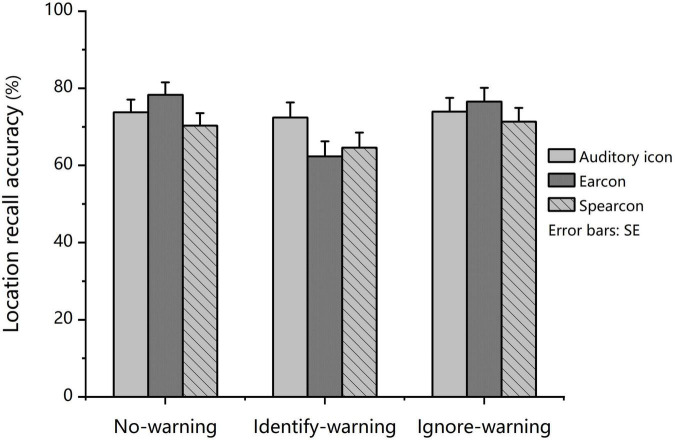
*Experiment 2:* Mean location recall accuracy in the no-warning, identify-warning, and ignore-warning conditions for the auditory icon, earcon, and spearcon groups.

A mixed-design 3 × 3 factorial ANOVA was performed on the effects of task condition and auditory warning type on red square location recall accuracy. The result revealed a main effect for task condition, *F* (2, 138) = 11.631, *p* < 0.001, partial η^2^ = 0.144. In addition to the main effect, a significant two-way interaction was found between task condition and auditory warning type (see Figure 4), *F*(4, 138) = 3.302, *p* = 0.016, partial η^2^ = 0.087.

Further simple effect analysis was conducted by using a *post hoc*-test with Bonferroni correction. The results indicated that for the auditory icon and spearcon groups, the mean location recall accuracy did not differ across the three task conditions. However, for the earcon group, the mean location recall accuracy in the identify-warning condition was significantly lower than that in the other two conditions (*p* < 0.001). For the identify-warning condition, the mean location recall accuracy was in the order: auditory icon group > spearcon group > earcon group; however, the differences were not significant. For the ignore-warning condition, the mean location recall accuracy for the three groups was not significantly different either.

**FIGURE 5:**
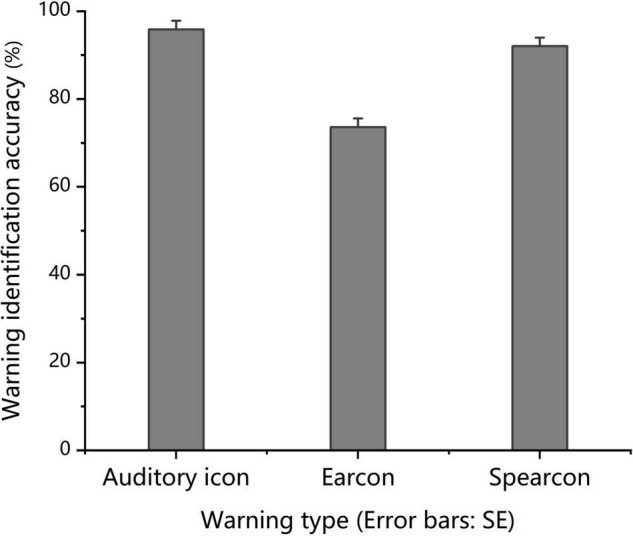
*Experiment 2:* Mean warning identification accuracy in the identify-warning condition for the auditory icon, earcon, and spearcon groups.

One-way ANOVA results indicated significant differences in the mean identification accuracy of the three groups, *F* (2, 69) = 35.701, *p* < 0.001, partial η^2^ = 0.509. The identification accuracy of earcons was significantly lower than those of auditory icons and spearcons (*p* < 0.001), but no significant difference was observed between auditory icons and spearcons as shown in Figure 5.

Spearman correlation analysis was conducted to further confirm whether the results were related to the learnability of auditory warnings. No significant correlation was found between learnability and mean location recall accuracy when the warnings were identified (*r* = 0.141, *p* > 0.05) or ignored (*r* = −0.082, *p* > 0.05). However, the mean warning identification accuracy was found to be significantly correlated with the learnability (*r* = 0.559, *p* < 0.001), which was consistent with the finding in Experiment 1.

### Discussion

Experiment 2 results showed that auditory icon identification had no significant interference on location recall task and had the highest identification accuracy among the three types of warnings. By contrast, earcon identification had significant interference on location recall task and the lowest identification accuracy. These results were consistent with experiment 1. The identification of earcons may require more working memory than that of auditory icons and spearcons. Participants may not be able to identify the earcons within their residual capacity of available resources, thereby leading to a competition with the location recall tasks for limited resources and resulting in low accuracy for the latter (Wickens, 2008).

**FIGURE 6:**
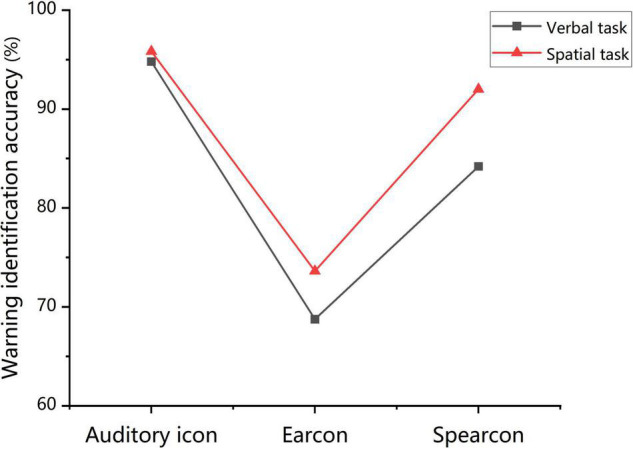
Mean warning identification accuracy for the auditory icon, earcon, and spearcon groups in the concurrent verbal and spatial working memory task.

**FIGURE 7:**
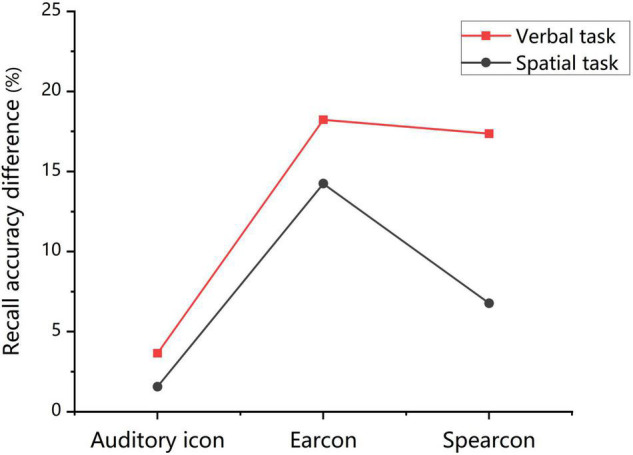
Mean recall accuracy difference (*M*_acc for ignore–warning_ - *M*_acc for identify–warning)_ in the ignore-warning and identify-warning conditions for the auditory icon, earcon, and spearcon groups in the verbal and spatial working memory task.

The results of warning identification accuracy in Experiment 2 were consistent with those in Experiment 1. The identification accuracy was the highest for auditory icons, followed by spearcons and earcons. The results of both experiments were combined for a rough comparison. The findings showed that the overall performance of warning identification in the concurrent verbal task was worse than that in the concurrent spatial task, especially for the spearcons (see Figure 6). In the concurrent verbal task, the identification accuracy of auditory icons was significantly higher than that of spearcons. Meanwhile, no significant difference between auditory icons and spearcons was found in the concurrent spatial task. The correlation between learnability and warning identification performance for verbal tasks was lower than that for spatial tasks. This indicated that the accuracy of warning identification was weakly affected by learnability but greatly influenced by the concurrent verbal task, compared with that in the concurrent spatial task. Recent studies found that concurrent verbal tasks would reduce the ability of participants to identify the spearcons (Davidson et al., 2019), which is consistent with the present results. Furthermore, the impact of identifying warnings on working memory was roughly analyzed by comparing the recall accuracy difference in the ignore-warning and identify-warning conditions. Although identifying auditory warnings (e.g., earcons) may interfere with spatial tasks, they consistently had a greater impact on verbal tasks, especially spearcons (see Figure 7). Therefore, we further speculated that warning identification had a greater impact on the overall performance of the verbal working memory task than that of the spatial working memory task. Meanwhile, the verbal working memory task had a greater impact on the overall performance of warning identification than the spatial working memory task. The greatest variation in spearcons might be related to their speech features. Early studies suggested that non-speech sounds did not interfere with working memory (Salamé and Baddeley, 1987), though subsequent work revealed that this depended on other factors of auditory information.

However, it is important to acknowledge that some of the comparisons and discussions here are made across two experiments. Further verification is needed, due to the possible existence of the failure of random assignment and the influence of uncontrolled changes.

## General Discussion

### Different Impacts of Three Types of Auditory Warnings on Working Memory

The impact of different types of auditory warnings on verbal and spatial working memory was examined. Current findings showed that identifying auditory icon warnings did not interfere with verbal and spatial working memory; however, identifying earcon warnings worsened participants’ performance on both verbal and spatial recall tasks, whereas identifying spearcon warnings only affects verbal recall tasks. These results showed that identifying different types of auditory warnings has different effects on verbal and spatial working memory.

Auditory icon warnings did not interfere with verbal and spatial working memory, either when the warnings were ignored or identified. Several usability studies indicated that auditory icons have better intuitiveness, learnability, and memorability than earcons (Garzonis et al., 2009; Isherwood and McKeown, 2017; Amer and Johnson, 2018). Our current work also indicated that auditory icons had good learnability. Participants may have completed a series of actions (switching attention, analyzing the acoustical input and then mapping the sound onto the linguistic token) with fewer working memory resources. Hence, the auditory icons did not interfere with the performance of working memory tasks because the identification of auditory icons may have placed less demand on the participants’ working memory than that of earcons and spearcons. The participants may have identified the auditory icon warnings within their residual capacity of available resources, thus preserving high accuracy at recall tasks (Wickens, 2008).

Identifying earcon warnings significantly affected the performance of both verbal and spatial working memory tasks. Earcons are synthetic sounds and are not directly related to the objects, events, or concepts they represent (Bonebright and Nees, 2007; Ludovico and Presti, 2016). The abstractions caused by the lack of semantic connection between earcons and representational events increase the difficulty of users’ understanding and memory. The participants’ efforts to remember the earcon warnings or to map the earcon sounds to the warning semantics took up additional resources, resulting in interference on working memory. Furthermore, the discovered effects of earcon identification on spatial working memory tasks supported the existence of a domain common resource in the mental process of verbal and visual space (Egeth and Kahneman, 1975; Barrouillet et al., 2004). The present study verified that verbal and visual spatial activities share a common general domain resource pool to a certain extent. The generality of these results was strengthened by other findings. For example, visual recall performance (memory for colored disks) was interfered with by simultaneous non-visual activities, such as tone-pitch recognition (Stevanovski and Jolicoeur, 2007). Increasing the cognitive load of concurrent spatial processing tasks reduced the performance of verbal recall tasks (Portrat et al., 2009; Vergauwe et al., 2010).

Identifying spearcon warnings only interfered with the verbal working memory. Given the worse accuracy for spatial recall task in no-warning or ignore-warning than for verbal recall task, the overall difficulty or the resources required to complete it for the location recall task might be higher than those for the serial recall task. However, the results showed that identifying spearcon warnings only affected the performance of the serial recall task, but not that for the location recall task. This finding indicated that the general resources occupied by spearcon identification were insufficient to seriously impair spatial task performance, but spearcon identification might have caused a significant domain-specific interference on the verbal working memory. Given that spearcons are a hybrid auditory display between speech and non-speech (Jeon, 2015), this phenomenon may be related to their speech characteristics. These results are consistent with the prediction of the working memory model theory (Baddeley and Logie, 1999). When combined with verbal activities, the performance of verbal memory task is worse than that of non-verbal memory task (Logie et al., 1990; Meiser and Klauer, 1999; Bayliss et al., 2003). One possible reason is that in the pronunciation control part of the phonological loop, the participants might “convert” the digits of the text form into speech codes through subvocalization and make them access the phonological storage device during the presentation of the digital stimulus items, thus causing interference.

The above findings are also consistent with the multi-resource theory (Wickens, 2002). The perception modality of concurrent tasks differed. The spatial location recall task used the visual modality. In the serial recall task, the participants might memorize the digits by articulatory rehearsal, and the visual and auditory modalities might be employed. Thus, the serial recall task competed with the identification of auditory warnings for the same limited pool of auditory modality resources (Wickens, 2002). Furthermore, the serial recall task required an additional stage of phonological processing to convert visual text into vocalized speech, thereby increasing verbal working memory load. Meanwhile, the identification of auditory warnings which involved mentally mapping sounds to specific linguistic tags, also required a verbal working memory load. Hence, the two tasks were competing for the same resources, thus reducing the resources available for simultaneous processing of serial recall task. The domain-specific interference of identifying auditory warnings in the task of verbal working memory may also be explained by some research findings. Cowan and Morey (2007) found that the domain-specific effect of working memory was more significant in the encoding stage than in the maintenance stage. It was possible that verbal tasks interfered with both encoding and maintenance of verbal information, and visuospatial tasks interfered only with the maintenance. Additionally, verbal information was found to be maintained by two independent mechanisms: attentional refreshing and articulatory rehearsal (Hudjetz and Oberauer, 2007; Camos et al., 2009). Vergauwe et al. (2010) suggested that this phenomenon occurred because both mechanisms were interfered with by verbal processing, whereas the spatial task interfered only with attentional refreshing. Nevertheless, further research is needed for verification.

### Are Auditory Warnings Ignorable?

An interesting finding here was that for the three types of warnings, no interference was observed when they were ignored by participants. This finding seems to be inconsistent with the irrelevant sound effect (Macken and Jones, 2003; Hughes et al., 2007; Macken et al., 2009). However, recent research showed that verbal recall tasks were only disrupted by irrelevant speech, but not by the presence of music or noise. The findings may be explained by a functional dissociation between working memory for phonological and non-phonological auditory items (Kattner and Meinhardt, 2020). The researchers found that for melodic and rhythmic alarms, the interference on verbal tasks was observed only when the alarm was identified (Lacherez et al., 2016). This may be related to attentional capture. Participants have to divide their attention (or switch attention) between the two tasks when identifying an auditory warning, which together with the process of identifying the warning takes up limited general resources and leads to interference. In turn, there may be no such processes when the warning was ignored, and therefore the recall task performance stayed untouched. Lacherez et al. (2016) found that when the auditory warning was ignored, melodic and rhythmic warnings did not affect the recall task, while a spoken non-word phrase warning did. These findings indicated that the effect in the ignore-warning condition may be related to the warning type. Therefore, the current results cannot be completely attributed to attention capture and may need to be interpreted from a deeper resource perspective. In fact, attention may be regarded as a general-purpose pool of limited resources (Vergauwe et al., 2010). Thus, it seems that the impact of warning on working memory may ultimately be due to resource occupancy and interference; once the occupied resources reach the threshold, the performance of concurrent tasks may be affected (Wickens, 2008). Ignoring the three types of warnings did not affect working memory, which may be due to the resources occupied by the action of ignoring warnings did not reach the interference threshold. Additionally, previous studies have found that music containing many rhythms or pitch variations is more disturbing than that with many legato passages (Klatte et al., 1995). This finding was consistent with the changing state hypothesis (Jones et al., 1992; Lecompte, 1995), which indicates that speech affects the performance of working memory tasks mainly because irrelevant sound stimuli are altered before and after stimulus entities. Banbury et al. (2001) suggested that acoustical changes are the main cause of interference, which is adjusted by the sensory organization of sound (e.g., flow). Repeated sounds, tones, or speech would not cause interference (Banbury et al., 2001). The duplex-mechanism account holds that changing-state stimuli do not capture attention; rather, the pre-attentive and obligatory processing of the order of the changing stimuli (warning sounds) conflicts with the serial rehearsal of the to-be-remembered stimuli (serial recall tasks) (e.g., Jones et al., 1996; Hughes and Jones, 2005). The current results might be related to the length of the warning sound materials. In this work, the length of sounds was approximately 1 s, which was relatively short and has small acoustic variability. The cue generated by the changing-state stimuli to order that conflicts with the processing of order in the concurrent task was less. A longer sound can have more acoustic changes. Future research should use different lengths of warning sounds to determine whether the impact of irrelevant warning sounds (i.e., ignored warnings) on working memory is related to the duration of the sounds.

### Practical Implications

The results of the present study provide important preliminary evidence that the perception and identification of learned auditory warnings (earcons and spearcons) interfere with working memory, at least in the laboratory task. However, we have to recognize that this property of capturing attention of warnings and the potential to interfere with processing represents the flip-side of the property of auditory warnings, which is often held to be their greatest asset (Ljungberg and Parmentier, 2012). One might think that people want auditory warnings to break in on other tasks. Nevertheless, our present research demonstrated that there may be additional costs for individuals who need to hold information in their memory. Listeners may not realize that listening to and identifying warnings may cause them to forget or ignore details that might be important to their current work. Therefore, people in work environments that use multiple auditory warnings should consider the mental load required in the execution of duties and how these might be affected by such distractions. The findings suggest that people might need to be reminded to pay attention not only to the effectiveness of auditory warnings but also to their potential impacts when designing auditory warnings, especially given the possible overuse of auditory warnings in high workload working environments.

The three types of auditory warnings did not interfere with working memory when ignored by the participants. This news appears encouraging because it suggests that learned warning sounds are at least negligible when the listener is informed to ignore them. Only the effort of identification causes the interference. Familiarity with warnings does not lead to involuntary or compulsory processing, or the resource occupancy generated when ignoring the warning does not reach the threshold of interfering with concurrent tasks. The operators engaged in a high-priority task may be able to prioritize their work over warning identification when they are willing to disregard the auditory warnings. Alternatively, operators can set the priority of their work to be higher than that of identifying auditory warnings, thereby reducing the potential problems of auditory warnings to some extent.

The identification of auditory icon warnings did not interfere with either verbal or spatial working memory and had the highest identification accuracy among the three types of warnings. Extensive work on the development of new auditory warnings for the medical device safety standard IEC 60601-1-8 demonstrates in many different ways (audibility, learnability, localizability, etc.) that auditory icons work well as auditory warnings in simulated clinical settings (Edworthy et al., 2017, 2018; Bennett et al., 2019). They found that anesthesia providers more correctly and quickly identified auditory icon alarms than standard earcon alarms, and participants were more likely to perceive lower fatigue and task load when using auditory icon alarms (McNeer et al., 2018). Therefore, considering the potential impact of identifying auditory warnings on working memory, auditory icon warnings may be a good choice for auditory warnings.

It is worth noting that identifying earcon warnings had the largest interference on working memory and the lowest identification accuracy among the three groups. The relationship between earcon and meaning is not based on environmental experience. Users need to learn how earcons relate to events or concepts (Amer and Johnson, 2018). Studies have found that earcons are inferior to spearcons in terms of learnability and identification accuracy (Palladino and Walker, 2007; Dingler et al., 2008; Walker et al., 2013) and have worse intuitiveness, learnability, and memorability than auditory icons (Garzonis et al., 2009; Isherwood and McKeown, 2017; Amer and Johnson, 2018). Therefore, it might be necessary to avoid the use of earcons as auditory warning signals, especially in high-load environments.

Identifying spearcon warnings interfered with verbal working memory, but not with spatial working memory. Therefore, spearcons may be an appropriate choice for warning signals in environments involving spatial working memory tasks. However, given the domain-specific interference of identifying spearcons on verbal working memory tasks, it may be necessary to avoid using spearcons as warning signals in environments involving verbal tasks. Although many other factors must be considered, the current results provide useful guidelines for the selection and design of auditory warnings.

### Limitations and Further Research

Many processes are involved in warning identification. Before identifying the presented warning, participants need to capture the entire warning sequence in their working memory and possibly need to mentally replay this warning to instill it in their memory. Some of the issues mentioned by Lacherez et al. (2016) were in agreement with the present study. The observed interference could be caused by auditory or phonological interference, or by analyzing the acoustical input (decoding the sound) and mapping the sound to linguistic tags (warning name). In the response selection, the participants were asked to identify the warning and press a specific key. This response might have affected the recall performance of the next trial (Lacherez et al., 2016). According to the theory of working memory model (Baddeley, 2000a), interference occurs at the encoding stage, that is, during item presentation rather than at the maintenance stage. Sounds affect the information storage in the phonological storage device. Visual stimuli (memorization items) are rehearsed into the form of phonemes and stored in the phonological storage device. Auditory phonemes that are automatically entered into the phonological storage device are confused with those converted from visual stimuli, thus resulting in interference. However, the object-oriented episodic record model (Jones and Macken, 1995) emphasizes that sounds weaken the performance of series recall by destroying sequential information and series rehearsal processing. It holds that sounds can cause interference in both the presentation and maintenance stages of memorization items. Therefore, many questions remain concerning the precise locus of interference. In our ongoing work, we consider the above theoretical hypothesis, and systematically manipulate the timing of warning sounds within relevant research paradigms to further elucidate the location of interference.

Novelty sounds (often called “deviant sounds”) capture attention, and the capturing of attention is a property of auditory warnings. In the present study, participants heard a set of four learned warning sounds in each condition, and the sounds were presented in random order. A learned auditory warning, once associated with a piece of information, may be more difficult to ignore than a seemingly random pattern; however, the participants’ repeated exposure to the warning sounds in the current study may reduce their elements of surprise, making the sounds easy to ignore and reducing the deviation effect (Cowan, 1995; Titova and Näätänen, 2001), and therefore performance stays unaffected. Nevertheless, whether the warning sounds in our experiments are easy to ignore might require further verification. Future research could take the role of attention capture into account and use deviant sound as an auditory warning to ensure the surprising attribute of warning sounds, and to further verify whether various types of warning sounds are ignorable.

The present research has some other limitations. First, the comparison focused on the impact of three types of auditory warnings (auditory icons, earcons, and spearcons) on working memory. In practical application, auditory warnings may consist of linguistic sounds with semantics, which may cause some high-order interference but might be easily recognized. Spearcons are a compromise between non-speech stimuli and full speech stimuli (Wolters et al., 2012). They may increase the amount of processing required compared to full speech auditory warnings that are more easily recognized. To comprehensively clarify the effects of various auditory warnings on working memory, future work should employ an identification warnings task, triggered by semantically related full speech, to determine whether speech warnings create similar interference to spearcons in terms of their effects on verbal and spatial recall tasks.

Second, it is important to acknowledge that we discuss the difference in the interference of auditory warnings for verbal and spatial working memory using data obtained across experiments. As mentioned in the discussion of Experiment 2, further verification is required due to the possible failure of random assignment and the influence of some uncontrollable factors. The current results are insufficient to conclude that auditory warnings create more interference with verbal working memory than with spatial working memory. The impact of auditory warnings on verbal vs. spatial recall tasks should be compared within one experiment in future work.

Third, the participants recruited in this study were college students. Given that working memory ability is related to age, and the age groups of operators in working environments may be various. The results should be further verified in other age groups in future studies.

## Conclusion

The purpose of this research was to investigate the impact of different types of auditory warnings on the performance of recall tasks involving verbal and spatial working memory. The results indicated that identifying auditory icon warnings did not interfere with either verbal or spatial recall tasks; however, identifying earcon warnings worsened participants’ performance on both verbal and spatial recall tasks, and identifying spearcons affected verbal recall tasks. These findings could raise concerns about the potential problems of using auditory warnings in working environments and provide useful guidelines for the selection and design of auditory warning signals. Further research is required to address the limitations of the present study, to elucidate the location of interference, and add the attributes of capturing attention and warning types to make warning sounds more ecologically valid, as well as to extend the comparative investigation to a more comprehensive scope.

## Data Availability Statement

The raw data supporting the conclusions of this article will be made available by the authors, without undue reservation.

## Ethics Statement

The studies involving human participants were reviewed and approved by the Institutional Review Boards of Zhejiang Sci-Tech University. The participants provided their written informed consent to participate in this study.

## Author Contributions

ZY and SM contributed to conception and design of the experiments. ZL recruited the participants and conducted the experiments. ZL and ZY performed the statistical analysis and wrote the manuscript. SM, ZY, and HL supervised the whole study. All authors contributed to manuscript revision, discussion, and approved the submitted version.

## Conflict of Interest

The authors declare that the research was conducted in the absence of any commercial or financial relationships that could be construed as a potential conflict of interest.

## Publisher’s Note

All claims expressed in this article are solely those of the authors and do not necessarily represent those of their affiliated organizations, or those of the publisher, the editors and the reviewers. Any product that may be evaluated in this article, or claim that may be made by its manufacturer, is not guaranteed or endorsed by the publisher.
